# The Effects of Sporting and Physical Practice on Visual and Kinesthetic Motor Imagery Vividness: A Comparative Study Between Athletic, Physically Active, and Exempted Adolescents

**DOI:** 10.3389/fpsyg.2021.776833

**Published:** 2021-11-23

**Authors:** Mohamed-Ali Dhouibi, Imed Miladi, Ghazi Racil, Sabra Hammoudi, Jeremy Coquart

**Affiliations:** ^1^Laboratory of Clinical Psychology: Intersubjectivity and Culture, Faculty of Humanities and Social Sciences, University of Tunis, Tunis, Tunisia; ^2^Higher Institute of Sport and Physical Education of Ksar Saïd, University of Manouba, Tunis, Tunisia; ^3^Research Unit (UR17JS01) Sport Performance, Health & Society, Higher Institute of Sport and Physical Education of Ksar Saïd, University of Manouba, Tunis, Tunisia; ^4^Tunisian Research Laboratory Sports Performance Optimization, National Center of Medicine and Science in Sports (CNMSS), Tunis, Tunisia; ^5^Univ. Lille, Univ. Artois, Univ. Littoral Côte d’Opale, ULR 7369 - URePSSS - Unité de Recherche Pluridisciplinaire Sport Santé Société, Lille, France

**Keywords:** visual motor imagery, kinesthetic motor imagery, physical education, sport practice, children

## Abstract

The interest of motor imagery practice on performance and motor learning is well-established. However, the impact of sporting and physical practice on motor imagery vividness is currently unclear, especially in youth. Two-hundred-and-forty adolescents were recruited to form different groups. For each age group (age-group 1, A-G1 with 13years≤age≤14years 6months vs. age-group 2, A-G2 with 14years 6months<age≤16years), 40 athletes, 40 active adolescents, and 40 exempted were recruited (20 girls and 20 boys in each category). Movement Imagery Questionnaire-Revised Second version (MIQ-Rs) was used to assess the Visual Motor Imagery (VMI) and Kinesthetic Motor Imagery (KMI) vividness. Results show that VMI is more evoked and more vivid than KMI (*p*<0.001). Athletes had greater VMI and KMI than active and exempted groups (*p*<0.001), and the active group also performed higher VMI and KMI than the exempted group (*p*<0.001). Subjects from A-G2 had greater motor imagery than subjects from A-G1, and boys had better motor imagery than girls. Conclusion: the present results show that sport and physical education engagement is associated with enhanced motor imagery vividness, especially in VMI. Moreover, older adolescents evoke clearer images than younger adolescents, and boys have greater imagery ability than girls. Therefore, teachers and coaches should consider age and gender when developing this cognitive skill when learning, in physical education classes and sports clubs.

## Introduction

Motor imagery (MI) is an active mental representation of a resynthesized motor experiences without any concomitant movement (Dickstein and Deutsch, 2007; Guillot and Collet, 2013; Williams et al., 2013; Vasilyev et al., 2017). According to Smith et al. (2007), MI training is known to improve sporting performance [for review: Nobuaki et al. 2012; Behrendt et al. 2021]. Some studies have shown that using MI immediately prior to movement execution permits to enhance motor actions and thus performance in various sports such as tennis (Robin et al., 2007), dart throwing (Nordin and Cumming, 2007), golf (Short et al., 2002; Williams et al., 2013), and soccer (Sariati et al., 2021). Indeed, MI activates the mental representations responsible for actual movement (Murphy et al., 2008). Through this pre-activation, the neurons responsible for movement are likely to be more prepared to correctly activate during actual movement execution. In addition to improving motor action, MI seems to enhance the self-confidence (Gregg et al., 2005), concentration (Sirigu et al., 1996), motivation (Yalcin and Ramazanoglu, 2020), focused attention (Ghodosi, 2014; Puyjarinet, 2019), reduce anxiety (Perna et al., 1995), and depression (Mousavi and Meshkini, 2011; López-Pérez et al., 2018). MI can have an internal (i.e., athlete visualizes him/herself as doing the motor task) or external (i.e., the athlete visualizes him/herself from third-person’s perspectives) perspective and can be visual (VMI, which involves visualization of action) or kinesthetic (KMI, which implies somesthetic sensations elicited by action; Decety et al., 1991; Giacobbi et al., 2003; Dickstein and Deutsch, 2007). However, the individual capacity to elicit such MI (i.e., MI vividness) is not universal and varies within and between subjects (Zabicki et al., 2019). So, for the past few decades, MI has been gaining momentum in sports, mainly in adults leaving a gap in the use of the MI by children. Therefore, better understanding the effects of MI in children is important given that the brain is developing in childhood, so assuming MI has the same effect as in adults may lead to erroneous conclusions being drawn on its effectiveness in youth. The few studies that have focused on MI and cognitive skills in children and adolescents suggested an age effect (Munroe-Chandler et al., 2007; Caeyenberghs et al., 2009; Hicheur et al., 2017; Souto et al., 2020) but reported equivocal findings about the gender, since some studies have noted that there are significant differences between boys and girls (Munroe-Chandler et al., 2007), while other studies have shown the opposite (Hall et al., 2009). Thus, it seems important to continue to study the effect of age on MI vividness in children. Accordingly, Smith and Wakefield (2013) and Spruijt et al. (2015) have noted that MI emerges around the age of 5–7years and stabilizes between adolescence and adulthood. However, Choudhury et al. (2007a,b) have shown that MI continues to develop between adolescence and adulthood. Few studies have investigated individual differences in MI vividness across samples of athletes, with fewer still considering youth athletes (Simonsmeier et al., 2017). However, it has been shown that young competitive athletes showed higher scores on MI vividness than non-athletes (Di Corrado et al., 2020). Thus, the practice of sports also seems to affect the MI vividness. Nevertheless, to our knowledge, there are no studies that have investigated the relationship between MI and sport and/or physical education (PE) to discern whether one or both of these physical practices can enhance MI ability through its vividness; as noted by Isaac and Marks (1994), who showed that vividness has often been used as measure of imagery ability amongst athletes with self-report questionnaires. Gammage et al. (2000) and Dey et al. (2012) consider that sport, as a structured practice, requires considerable physical and mental effort, while PE, which is not limited to structured contexts, requires less physical and mental effort compared to sport. In addition, Johnson et al. (1981) argue that cooperative learning situations are more effective than competitive situations. Therefore, Tobin et al. (2013) and Guerrero and Munroe-Chandler (2018) think that it is reasonable to assume that children’s MI in a PE setting would be more developed. Accordingly, the present study sought to evaluate the effect of sport and PE on MI vividness by raising the following questions: (i) would sport, as an institutionalized and deliberate physical practice, affect MI vividness? (ii) if so, could it have more influence than PE? (iii) should developing a vivid motor image depend on physical practice (considered as an environmental factor) or rather and quite simply on neural and brain maturity? (iv) would the imaging vivacity be influenced by gender and age? (v) at the age of adolescence, would VMI prevail on KMI, or vice-versa?

## Materials and Methods

### Participants

For each age group (age-group 1, A-G1 with 13years≤age≤14years 6months vs. age-group 2, A-G2 with 14years 6months<age≤16years), 40 athletes, 40 active adolescents, and 40 exempted adolescents were recruited (20 girls and 20 boys in each category). The athletes were adolescents who practice football, athletics, tennis, judo, and swimming, and they engaged each year in training in sport club for at least the last 4years (≈ 190h each year), in addition to PE sessions (≈ 40h each year). The active participants included those who only attend PE sessions at high-school yearly for at least the last 4years (≈ 40h each year). The exempted group included adolescents who did not participate in any physical activity outside of school (in sports clubs) or within school (in PE or sports classes). Any activities practiced outside structured framework (sports clubs or schools) were not be taken into account. According to the ONS (2010), exempted students represent more than 5% of students in middle and high school. Medical staff checked that exempted students had to (i) have a medical certificate stating that they were unfit to practice a physical activity or sport, (ii) not be unfit because of a motor or mental disorder or disability, and (iii) have been exempted for at least the last 4years. All participants belong to the mid-adolescent age group, which is characterized by the increase of the abstraction cognitive capacity (Devernay and Viaux-Savelon, 2014). Taken account of this developmental perspective, we have divided our population into two age groups, as suggested by Puyjarinet et al. (2020) to verify the impact of age factor on MI vividness. To avoid possible effect on KMI or VMI, all participants had to be right-handed [for review: O’Regan and Serrien (2018)] and had normal or corrected-to-normal vision [for review: Omar et al. (2017)]. Out of respect for research ethics, written informed consent was requested from the legal tutors/parents of each participating child following the explanation of the study protocol. The characteristics of population are presented in Table 1.

**Table 1:** Distribution of population by physical activity level, age, and gender.

Groups	Age groups	Age ranges	Gender	*n*	Age
Athlete	A-G1	13.0–14.5 y	Boys	20	13.98±0.40 y
			Girls	20	14.02±0.41 y
	A-G2	14.5–16.0 y	Boys	20	15.27±0.42 y
			Girls	20	15.20±0.17 y
Active	A-G1	13.0–14.5 y	Boys	20	14.10±0.35 y
			Girls	20	13.85±0.47 y
	A-G2	14.5–16.0 y	Boys	20	15.31±0.36 y
			Girls	20	14.99±0.29 y
Exempted	A-G1	13.0–14.5 y	Boys	20	13.78±0.46 y
			Girls	20	13.43±0.23 y
	A-G2	14.5–16.0 y	Boys	20	15.46±0.34 y
			Girls	20	15.01±0.31 y

*A-G1 with 13years≤age≤14years 6months; A-G2 with 14years 6months<age≤16years; n: sample size*.

### Procedure

Initially, all participants completed three non-verbal neuropsychological tests: the Corsi block task to measure the spatial short-term memory capacity (Corsi, 1972), the Revision Visual Retention Test (RVRT) to evaluate visual memory and visuospatial functions in their various aspects “visuomotor, visuospatial, and visuoconstructive” (Benton, 1974), and the Test Of Nonverbal Intelligence – second edition (TONI 2) to evaluate the nonverbal intelligence (Brown et al., 1990). None of the participants scored below expected norms, hence showing normal intellectual functioning (see Table 2). Then, the participants completed the Movement Imagery.

**Table 2:** Results of the non-verbal neuropsychological tests.

Age groups	Gender	Brown non verbal intelligence test		Corsi block task test	Benton visual retention test		
		IQ	Percentile	M±SD	Adm. B (correct score) M±SD	Adm. C (error score) M±SD	Adm. D (correct score) M±SD
A-G1	Boys	117±7	87	7.00±0.69	7.26±0.58	0.40±0.17	7.22±0.59
	Girls	116±8	86	6.87±0.70	7.24±0.58	0.50±0.20	7.20±0.53
	Total	116±8	86	6.94±0.70	7.25±0.58	0.42±0.19	7.21±0.56
A-G2	Boys	118±8	88	6.60±0.74	7.21±0.68	0.42±0.19	7.22±0.48
	Girls	118±7	88	6.37±0.68	7.23±0.40	0.50±0.21	7.11±0.52
	Total	118±8	88	6.49±0.71	7.22±054	0.55±0.20	7.17±0.50

*A-G1 with 13years≤age≤14years 6months; A-G2 with 14years 6months<age≤16years; IQ, Intellectual quotient; Adm, Administration mode; M, Mean; SD, Standard deviation*.

Questionnaire-Revised Second version (MIQ-Rs; Gregg et al., 2008) in its valid French translated version (Loison et al., 2013). The 14 items of this questionnaire are divided into seven items to evaluate VMI and seven other items to evaluate KMI. The tasks to be mentally imagined involve the upper limb, the lower limb, the body as a whole, and tasks in daily life. For each item, the experimenter reads to the participant the description of the task to be carried out. Then, the adolescent actually performs the task and is then asked to either visually or sensorially imagine it. The participant is asked to rate the ease or difficulty (from a 7-point Likert scale) with which he has executed each mental task. The score for each item thus ranged from 1 “very hard” to 7 “very easy.” Each participant obtained a mean score in each scale (VMI and KMI). Higher score means better MI ability with regard to the tested modality. All tests and the questionnaire were carried out before the start of training sessions for all athletes’ subjects, during PE sessions for active subjects and outside of school classes for exempt subjects.

### Statistical Analysis

All values were expressed in the form of means ± standard deviations. Before using the parametric tests, the normality of distribution was verified by the Shapiro–Wilk W-test test. The data were analyzed using the MANOVA multivariate variance analysis (2×3×2×2). Factors included two MI modalities (called Conditions: VMI and KMI), three activity-group levels (Athletes, Active, and Sedentary), two age-group categories (A-G1 and A-G2) and two gender-groups (Boys and Girls). When a significant *F* value was achieved, a Bonferroni *post-hoc* analysis was performed. Partial eta-squared (*η*^2^) was used to calculate the effect size (ES) for all ANOVAs. Cohen (1988) considered the values of 0.01, 0.06, and 0.15 as small, medium, and large cut-off point, respectively. For all paired comparisons, effect sizes were calculated and judged according to the following scale: ≤0.2, trivial; >0.2–0.6, small; >0.6–1.2, moderate; >1.2–2.0, large; and>2.0, very large (Hopkins et al., 2009). Statistical significance was set at *p*<0.05, and all analyses were performed with the Statistical Package for the Social Sciences (version 18.0, Chicago, United States).

## Results

The results indicated an effect of the main factors: “Condition” (*F*=890.21, *p*<0.001, *d*=1.94), “Group” (*F*=969.49, *p*<0.001, *d*=2.84), “Age” (*F*=38.16, *p*<0.001, *d*=0.40), and “Gender” (*F*=89.68, *p*<0.001, *d*=0.62). VMI was always greater than KMI (*p*<0.001, *d*=4.16), regardless of group, age, or gender (see Table 3). Athletes showed superiority in both VMI and KMI compared to active participants (*p*<0.001, *d*=3.50), who in their turn performed better than exempted subjects (*p*<0.001, *d*=2.73) as shown in Table 4. Boys and girls in A-G2 had a greater imaging capacity than adolescents in A-G1 (*p*<0.001, *d*=0.25) as shown in Table 5. Boys had greater imagery scores than girls in both VMI and KMI (*p*<0.001, *d*=0.38) as shown in Table 6. Moreover, interactions were found between condition, group, age, and gender: “Condition×Group” (*F*=5.17, *p*<0.01, *d*=0.21), “Condition×Age” (*F*=6.41, *p*<0.05, *d*=0.17), “Condition×Gender” (*F*=26.47, *p*<0.001, *d*=0.34), “Condition×Group×Age” (*F*=8.87, *p*<0.001, *d*=0.28), and “Condition×Group×Gender” (*F*=14.63, *p*<0.001, *d*=0.36).

**Table 3:** Sport and physical practice effect on motor imagery vividness.

Groups	Age groups	Gender	Motor imagery modalities		Cohen’s *d*
			Visual	Kinesthetic	
Athlete	A-G1	Boys	5.49±0.19^a^	4.25±0.20	6.36
		Girls	5.05±0.42^a^	4.41±0.49	1.40
	A-G2	Boys	5.65±0.10^a^	4.52±0.30	5.05
		Girls	4.99±0.36^a^	4.53±0.33	1.33
Active	A-G1	Boys	4.60±0.38^a^	3.95±0.19	2.16
		Girls	4.01±0.22^a^	3.42±0.39	2.24
	A-G2	Boys	4.71±0.29^a^	3.96±0.22	2.91
		Girls	4.43±0.14^a^	3.61±0.25	4.05
Exempted	A-G1	Boys	3.77±0.34^a^	3.16±0.07	1.98
		Girls	3.54±0.23^a^	3.13±0.05	2.46
	A-G2	Boys	4.02±0.28^a^	3.11±0.07	4.46
		Girls	3.99±0.38^a^	3.17±0.09	2.97

a *Significantly different between visual and kinesthetic motor imagery (p<0.001)*.

*A-G1 with 13years≤age≤14years 6months; A-G2 with 14years 6months<age≤16years*.

**Table 4:** Groups effect on motor imagery vividness (comparison of groups 2 by 2).

Motor imagery modalities	Age groups	Gender	Groups			Cohen’s *d*		
			Athlete	Active	Exempted	Athlete vs. Active	Athlete vs. Exempted	Active vs. Exempted
Visual	A-G1	Boys	5.49±0.19^a^	4.60±0.38^b^	3.77±0.34	2.96	6.24	2.30
		Girls	5.05±0.42^a^	4.01±0.22^b^	3.54±0.23	3.10	4.46	2.08
	A-G2	Boys	5.65±0.10^a^	4.71±0.29^b^	4.02±0.28	4.33	7.75	2.42
		Girls	4.99±0.36^a^	4.43±0.14^b^	3.99±0.38	2.05	2.7	1.54
Kinesthetic	A-G1	Boys	4.25±0.20^a^	3.95±0.19^b^	3.16±0.07	1.54	7.27	5.52
		Girls	4.41±0.49^a^	3.42±0.39^b^	3.13±0.05	2.24	3.67	1.04
	A-G2	Boys	4.52±0.30^a^	3.96±0.22^b^	3.11±0.07	2.13	6.47	5.21
		Girls	4.53±0.33^a^	3.61±0.25^b^	3.17±0.09	3.14	5.62	2.34

a *Significantly higher than the two other groups (p<0.001)*.

b *Significantly higher than the exempted group (p<0.01)*.

*A-G1 with 13years≤age≤14years 6months; A-G2 with 14years 6months<age≤16years*.

**Table 5:** Age effect on motor imagery vividness.

Motor imagery modalities	Gender	Groups	Age groups		Cohen’s *d*
			A-G1	A-G2	
Visual	Boys	Athlete	5.49±0.19	5.65±0.10^a^	1.05
		Active	4.60±0.38	4.71±0.29^b^	0.32
		Exempted	3.77±0.34	4.02±0.28^a^	0.80
	Girls	Athlete	5.05±0.42	4.99±0.36	0.15
		Active	4.01±0.22	4.43±0.14^a^	2.28
		Exempted	3.54±0.23	3.99±0.38^a^	1.43
Kinesthetic	Boys	Athlete	4.25±0.20	4.52±0.30^a^	1.06
		Active	3.95±0.19	3.96±0.22	0.05
		Exempted	3.16±0.07^a^	3.11±0.07	0.71
	Girls	Athlete	4.41±0.49	4.53±0.33^b^	0.29
		Active	3.42±0.39	3.62±0.25^a^	0.61
		Exempted	3.13±0.05	3.17±0.09^a^	0.55

a *Significantly higher than A-G1 (p<0.001)*.

b *Significantly higher than A-G1 (p<0.01)*.

*A-G1 with 13years≤age≤14years 6months; A-G2 with 14years 6months<age≤16years*.

**Table 6:** Gender effect on motor imagery vividness.

Age groups	Motor imagery modalities	Groups	Gender		Cohen’s *d*
			Boys	Girls	
A-G1	Visual	Athlete	5.49±0.19^a^	5.05±0.42	1.35
		Active	4.60±0.38^a^	4.01±0.22	1.90
		Exempted	3.77±0.34^a^	3.54±0.23	0.79
	Kinesthetic	Athlete	4.25±0.20	4.41±0.49^b^	0.43
		Active	3.95±0.19^a^	3.42±0.39	1.73
		Exempted	3.16±0.07^b^	3.13±0.05	0.49
A-G2	Visual	Athlete	5.65±0.10^a^	4.99±0.36	2.50
		Active	4.71±0.29^a^	4.43±0.14	1.23
		Exempted	4.02±0.28	3.99±0.38	0.09
	Kinesthetic	Athlete	4.52±0.30	4.53±0.33	0.03
		Active	3.96±0.22^a^	3.61±0.25	1.49
		Exempted	3.11±0.07	3.17±0.09^a^	0.74

a *Significantly higher than adolescents of the other gender (p<0.001)*.

b *Significantly higher than adolescents of the other gender (p<0.05)*.

*A-G1 with 13years≤age≤14years 6months; A-G2 with 14years 6months<age≤16years*.

## Discussion

The main purpose of the current study was to determine the effect of physical practice on MI vividness in adolescents. Subsequently, we sought to investigate the effect of age and gender on imaging vividness and to discern the visual vs. kinesthetic properties using the MIQ-Rs. The present results highlighted that VMI is largely more vivid, developed, and evoked than KMI. However, these differences become less important when analyzing the groups separately, suggesting that VMI owes its superiority to the group of athletes. Indeed, boys and girls (regardless of the age group) in the athletes group evoke VMI more frequently. These results align with the works of Voisin et al. (2011) and Yoxon (2012) who showed that VMI is generally considered as easier to evoke and to maintain, unlike KMI. According to Baddeley and Andrade (2000), this vividness depends on the richness of the representations recorded in the working memory that are capable of triggering, generating, maintaining, and transforming the imaging process. This was explained by Parker and Lovell (2012), who argued that MI vividness is related to its manifestation when processing information. This means that the evoked sensory modality is largely considered as determinant of the MI modality and vividness (a greater vivacity of VMI depends to a high visual activity). Hall and Fishburne (2010) asserted that the use of images, as proposed by coaches and teachers, could be dependent on the sport practiced. Moreover, the exercise load could also play an important role in determining the imagery modality, and thus represents a viable avenue for further research. Then, the results show that the athletes have MI vividness significantly better than active subjects, who, in their turn, were better than exempted counterparts. This suggests that sport and/or PE practice offers adolescents a variety of motor experiences, enabling them to develop their ability to effectively process numerous motor skills information. To explain this, biological and social environmental parameters must be considered. According to Parker and Lovell (2012) and Seiler et al. (2017), neurophysiology offers some explanations of the imagery vividness through the functional magnetic resonance imaging (fMRI) measurements, allowing the differentiation between expert adults (i.e., with long-term motor practice) with high imagery ability and novice adults with low imagery ability. Indeed, Meister et al. (2005) and Aglioti et al. (2008) have noted that motor expertise is associated with functional changes in the brain, and Wu et al. (2013) have indicated an increase in the activation of certain targeted brain areas in confirmed athletes. In this sense, Olsson and Nyberg (2010) and Zhang et al. (2018) reported that adults with better imagery skills would activate neural circuits and areas during mental simulation differently than those with poor imagery skills. Comparing the brain activity of athletes to that of novices, Olsson and Nyberg (2010) found that during imaging a high jump, the premotor cortex, and the cerebellum were activated in confirmed jumpers, while visual areas (upper occipital cortex) were activated in the novices. In the same way, Seiler et al. (2015) confirmed that the activation of neural networks differs depending on the IM modality: (KMI activates motor areas, internal VMI activates the inferior parietal lobule, and external VMI activates temporal areas, but not occipital). However, it should be noted that other studies have recorded equivocal results regarding the activation of neural circuits during MI [see: Seiler et al. (2017)]. On the other hand, some authors have noted that the social environment can be considered to be one of the main determinants of the ability to evoke vivid images. Parker and Lovell (2012) believe that many activities are becoming important in our environment and able to influence spatial skills, including imaging functions. Concordantly, several institutions participate actively in the development of this mental skill, such as school or sport clubs. Arvinen-Barrow et al. (2008) and Zhang et al. (2018) indicated that athletes more often require the use and the creation of more images with greater ease than novices, demonstrating why they have better imaging scores. In addition, Schack et al. (2014) argue that high congruence between the cognitive framework and the biomechanical demands of a task is a fundamental tenet for any expert. In contrast, the task representations in novices and players with low-level are reported to be less organized hierarchically and in discordance with the functional and biomechanical requirements of their sport. It seems that MI increases with physical activity participation (Guerrero and Munroe-Chandler, 2017) and the frequency of training sessions (Lotze and Halsband, 2006), distinguishing recreational from professional athletes. Parker and Lovell (2009) confirmed that more than three training sessions per week facilitated the use of more images. School may be considered as a social environment that can increase imagery ability in children by improving psychomotor skills, especially when teaching them how to use and develop MI skills, primarily during PE sessions (Galyean, 1983). Flusberg and Boroditsky (2011) noted that, in school, PE is the main discipline based on sensory-motor content providing a rich and essential ground for the development of MI. In this way, Hall and Fishburne (2010) claimed that the use of imagery by the students achieving higher marks in PE classes, could reveal similar results in the skill mastery reported in sport. So far, little research has been specifically devoted to the use of MI in PE (Goudas and Giannoudis, 2008). However, there is an abundance of available literature on MI conducted in the fields of motor learning (Holmes and Collins, 2001) and sport psychology (Morris et al., 2005), which could have potential applications in a PE context. Finally, Nobuaki et al. (2012) indicated that it is still difficult to elucidate the causes of differences in the ability to create vivid motor images between novices and athletes, and this is even more true when it comes to young athletes (Isaac and Marks, 1994), and thus, will require more detailed investigations. The current results show also that age plays an important role in the development of MI vividness, where subjects belonging to A-G2 possessed better imagery skills than their A-G1 counterparts (see Table 6). These findings corroborate those of Arvinen-Barrow et al. (2008), where the oldest group of synchronized skaters (18.5years) used imagery skill more than the middle age athletes (15.3years), who themselves employed more MI than the youngest (12.9years) age groups. Kosslyn et al. (1990), Mitra et al. (2016), and Souto et al. (2020) argued that imagery ability is subject to changes with maturity, and age would have an influence in this process. With age, and compared to other skills, the vividness of an image is subject to improvements in both visual and kinesthetic modalities (Parker and Lovell, 2012). This could be mediated by very large volumes of sporting or physical practicing, which may offer more opportunities to use MI, allowing low and high performers to be distinguished (Robert and Gould, 2003). On the other hand, Choudhury et al. (2007a) suggested that the development of the parietal cortex could explain the improvement of MI between adolescence and adulthood. Indeed, Gogtay et al. (2004) and Toga et al. (2006) highlighted a development of gray and white substances in the parietal cortex, which may reflect synaptic pruning and myelination during adolescence. Future studies are needed to determine the differential involvement of cortical circuits in MI in adolescents compared to adults (Choudhury et al., 2007a). Overall, the results of this study suggest that boys perform better than girls in MI. However, by examining groups separately, we found that this difference fluctuates from one group to another. In the athlete group, boys in both A-G show superiority only in VMI. While among the active group, boys in both A-G are better than girls in VMI and KMI. We found that, among the exempted group, a superiority of boys belonging to the A-G1 is better in both VMI and KMI, while exempted girls belonging to A-G2 were better in KMI only (see Table 6). These findings correspond to those of Habacha et al. (2014) and Campos and Lustres (2019) who noted that gender may play a role in the development of MI. Yoxon (2012) specifies that in children, differences in VMI between boys and girls could be the result of sports practice and motor experiences. Indeed, increasing participation in sports and motor events could increase, or at least facilitate, the use of MI. De Caroli and Sagone (2007), as well as Hoyek et al. (2009), reported that boys were better at forming a dynamic mental image of movements compared to girls who had more difficulty in preserving the temporal organization of an imagined movement. Gao et al. (2014) further suggested that game preferences during childhood have determining roles: in boys, they mainly develop the visuospatial capacity and body image; whereas in girls, they develop the ability of communication. Contrary, some previous studies have shown that gender has minimal influence on the use of MI (Hall, 2001). Munroe-Chandler et al. (2007) studied the content of MI in young athletes aged 7–14years. They found no gender effect in the overall ability to form mental images. It is likely that similar results would be obtained for the influence of age and gender on the use of images in PE, but this remains to be confirmed (Hall and Fishburne, 2010), highlighting that further research is needed.

## Conclusion

Until recently, much of the existing literature on the use of images in sport has focused on adult athletes, with a paucity of research in the use of images by children and young athletes (Miller, 2017). Thus, the present study sought to clarify MI and its relationship with sport and PE practice, especially in adolescents. Our results are important for athletes and coaches in relation to the best use of MI to enhance sport and PE performance and show that sport and physical activity engagement is associated with enhanced MI vividness, especially in VMI. Moreover, older adolescents evoke clearer images than younger adolescents, and boys have greater imagery ability than girls. Therefore, teachers, trainers, and instructors are encouraged to take into account the importance of this cognitive skill and give it special attention during the learning process. However, not taking into consideration the type of sport as well as its practice environment, and only evaluating the overall VMI modality without distinguishing the external one from the internal one, present limitations for this study. It is necessary to conduct further work in order to elucidate the causal impact of sport and PE on MI.

## Data Availability Statement

The raw data supporting the conclusions of this article will be made available by the authors, without undue reservation.

## Ethics Statement

The studies involving human participants were reviewed and approved by the University of Tunis. Written informed consent to participate in this study was provided by the participants’ legal guardian/next of kin.

## Author Contributions

All the authors designed and conceptualized the study, interpreted the data, commented on next versions of the manuscript, and read and approved the final manuscript. MD, IM, GR, and SH collected the data. MD wrote the first draft of the manuscript.

## Conflict of Interest

The authors declare that the research was conducted in the absence of any commercial or financial relationships that could be construed as a potential conflict of interest.

## Publisher’s Note

All claims expressed in this article are solely those of the authors and do not necessarily represent those of their affiliated organizations, or those of the publisher, the editors and the reviewers. Any product that may be evaluated in this article, or claim that may be made by its manufacturer, is not guaranteed or endorsed by the publisher.
